# The Effect of Visual Perceptual Load on Auditory Awareness of Social vs. Non-social Stimuli in Individuals with Autism

**DOI:** 10.1007/s10803-020-04587-0

**Published:** 2020-07-01

**Authors:** Julian Tillmann, Jyrki Tuomainen, John Swettenham

**Affiliations:** 1 Department of Psychology, Institute of Psychiatry, Psychology & Neuroscience, King’s College London, De Crespigny Park, Denmark Hill, London, SE5 8AF UK; 2 Department of Applied Psychology: Health, Development, Enhancement, and Intervention, University of Vienna, Vienna, Austria; 3 Speech, Hearing & Phonetic Sciences, Division of Psychology and Language Sciences, University College London, London, UK; 4 Department of Language and Cognition, University College London, London, UK

**Keywords:** Autism spectrum disorder, Auditory awareness, Attention, Perceptual load, Social stimulus

## Abstract

This study examined the effect of increasing visual perceptual load on auditory awareness for social and non-social stimuli in adolescents with autism spectrum disorder (ASD, *n* = 63) and typically developing (TD, *n* = 62) adolescents. Using an inattentional deafness paradigm, a socially meaningful (‘Hi’) or a non-social (neutral tone) critical stimulus (CS) was unexpectedly presented under high and low load. For the social CS both groups continued to show high awareness rates as load increased. Awareness rates for the non-social stimulus were reduced when load increased for the TD, but not the ASD group. The findings indicate enhanced capacity for non-social stimuli in ASD compared to TD, and a special attentional status for social stimuli in the TD group.

**Electronic supplementary material:**

The online version of this article (10.1007/s10803-020-04587-0) contains supplementary material, which is available to authorized users.

## Introduction

Autism Spectrum Disorder (ASD) is an early-onset neurodevelopmental condition that is associated with difficulties in social interaction and communication, the presence of restricted, repetitive and stereotypic behaviours and interests, and atypical sensory processing (DSM-5, American Psychiatric Association 2013). ‘Social brain’ accounts of ASD (Adolphs 2009) propose that the social and communicative impairments in ASD stem from altered functioning of a network of cortical and subcortical structures specialised in the processing of socially-relevant stimuli including eye gaze, faces, speech and biological motion (Pelphrey et al. 2011; Dawson et al. 2012; Annaz et al. 2012). These alterations are thought to affect a range of processes early on in development, including reduced attention to social stimuli, which in turn restrict the child’s exposure to typical social interactions and affecting subsequent social development (Dawson et al. 2004; Swettenham et al. 1998; Annaz et al. 2012). One of the earliest manifestations of atypical attention to social stimuli in ASD is the poor orienting to the human voice (Klin 1991). Whereas typically developing (TD) children show an attentional bias towards speech sounds from a very early age (Alegria and Noirot 1978; Jusczyk and Bertoncini 1988), children with ASD often do not exhibit such a preference (Klin 1991, 1992; Dawson et al. 1998; Kuhl et al. 2005). For example, as early as 12 months of age, infants with ASD show a diminished orientation to their own name (Osterling and Dawson 1994; Baranek 1999; Osterling et al. 2002) and to child-directed speech (Klin 1991, 1992; Kuhl et al. 2005). Infants at high familial risk for ASD who go on to develop ASD later in life are also less likely to orient to their name at 12 months of age (Zwaigenbaum et al. 2005; Nadig et al. 2007; Miller et al. 2017), and a preference for speech sounds at this age in high-risk infants is associated with fewer autistic-like behaviours at 18 months (Curtin and Vouloumanos 2013).

Atypical patterns of brain activation to speech stimuli have also been observed in individuals with ASD. Gervais et al. (2004) found decreased neural activation in speech- and voice-selective areas such as the superior temporal sulcus (STS) in response to vocal stimuli (speech and vocal stimuli) in individuals with ASD relative to TD controls. Using resting-state functional MRI (rsMRI), Abrams et al. (2013) demonstrated reduced intrinsic connectivity between voice-selective brain regions (i.e. posterior STS) and cortical structures involved in emotion and speech processing such as the ventral tegmental area, nucleus accumbens, amygdala, and orbitofrontal cortex. Studies using event-related potentials (ERPs) also suggest that children with ASD show differences in the amplitude of the P3, a brain component related to attention. When asked to listen passively to either speech or non-speech stimuli, a number of studies have observed smaller or absent P3a amplitudes in response to speech stimuli, but not to non-speech sounds, in children and adults with ASD compared to age-matched TD controls (Lepistö et al. 2005; Lepistö et al. 2007; Lepistö et al. 2006; Ceponiene et al. 2003; but also see Whitehouse and Bishop 2008). Taken together, the accumulated evidence indicates a lack of attentional bias towards speech sounds in individuals with ASD, which likely emerges early on in development and may contribute to more broader deficits in social communication. However, it remains unclear why individuals with ASD demonstrate atypical orientation to socially relevant auditory information, and in particular to speech. It could be a result of a more fundamental perceptual impairment in acoustic encoding of complex stimuli, as advocated by Mottron et al. (2006). Conversely, reduced attention to social stimuli might underlie this deficit in orienting, such that individuals with ASD assign fewer attentional resources to social stimuli than TD individuals (Swettenham et al. 1998; Klin et al. 2002).

To further investigate attention towards socially-meaningful auditory stimuli in ASD, the current study applied perceptual load theory (i.e. Load theory; Lavie 1995) to examine whether altering the attentional demands (i.e. perceptual load) of a visual task affects awareness of an unexpected speech sound differently in individuals with ASD compared to TD controls. According to load theory, successful selective attention (i.e. selecting task-relevant information over task-irrelevant information) depends on whether the perceptual load of the task exhausts an individual’s limited processing capacity; perceptual load being defined as the amount of task-relevant information to be processed, such as the number of items in a search display or the subtlety of a line discrimination (Lavie 1995). In a task of high perceptual load, all available capacity is fully consumed by the processing of task-relevant information, leaving insufficient processing capacity available to process task-irrelevant or distracting information. By contrast, in a task of low perceptual load, any spare capacity left-over from the less capacity-taxing processing will automatically and involuntarily spill-over to the processing of task-irrelevant stimuli (Lavie 1995).

The effect of perceptual load on attention in TD individuals however depends on the nature of the task-irrelevant stimulus presented: while neutral, non-social stimuli produce the predicted load-dependent effect, distracting stimuli of high social relevance (e.g. faces) continue to be processed at higher levels of perceptual load (Lavie et al. 2003; Neumann and Schweinberger 2008; Neumann et al. 2011; Thoma and Lavie 2013). This indicates that in TD individuals, processing of socially-meaningful stimuli does not depend on general capacity limits and instead proceeds in an automatic, mandatory fashion regardless of the level of load imposed on task-relevant processing (Lavie et al. 2003; Thoma and Lavie 2013). Applying Load theory to investigate these effects in individuals with ASD, Remington et al. (2012a, b) demonstrated that adults with ASD do not show the same automatic processing of faces in high load task conditions. Participants had to indicate whether a central target name, presented among other non-words, was female or male (e.g. Katie or John) whilst ignoring either congruent (same gender as target name) or incongruent (opposite gender as target name) distractor faces shown simultaneously in the periphery. While TD individuals demonstrated a high level of distraction irrespective of the perceptual load of the task (i.e. number of non-words presented simultaneously), processing of distractor faces in individuals with ASD was only evident at low, but not high levels of perceptual load. This was taken as evidence that faces do not automatically capture attention in adults with ASD to the same extent as in typical adults, where faces seem to have a ‘special status’ for attention and are processed regardless of their task relevance or the perceptual load of the task (Remington et al. 2012a, b).

In the current study, we sought to contrast load-dependent effects on attention for different types of auditory stimuli: a socially meaningful auditory stimulus vs. a neutral, non-social auditory stimulus by adopting an inattentional deafness task (Macdonald and Lavie 2011; Tillmann et al. 2015). Inattentional deafness relates to the phenomenon that most participants fail to notice an unexpected auditory stimulus on a critical trial when their attention is engaged by a relevant primary task yet report the presence of the same stimulus on a following ‘full attention’ control trial when told to ignore the primary task and instead pay attention to anything else. Previous studies suggest that for brief neutral, non-socially meaningful tones, rates of inattentional deafness are less affected by increases in visual perceptual load in individuals with ASD compared to TD individuals (Tillmann et al. 2015; Tyndall et al. 2018). Tillmann et al. (2015) for example asked children with and without ASD to make either low or high-load visual discriminations (i.e. making either a gross or subtle line discrimination), and on the last of seven trials, the critical trial, a brief unexpected neutral tone was presented simultaneously with the visual task display. Results indicated that for TD children, the rate of awareness reports on the critical trial was considerably lower at high perceptual load than at low perceptual load—increasing visual load induced inattentional deafness for the neutral tone. In contrast, rates of awareness for children with ASD were equally high in both high- and low-load conditions—visual perceptual load had no effect on auditory awareness rates in the ASD group (Tillmann et al. 2015). Using the same paradigm and identical load manipulations, Tyndall et al. (2018) replicated these findings in adults with ASD, showing higher awareness rates of a neutral tone at high perceptual load in adults with ASD relative to TD adults. In addition, the authors made preliminary attempts to examine the effects of visual perceptual load on awareness of a socially meaningful auditory stimulus (‘Hi’), yet without making specific predictions about load effects in individuals with ASD. Exploratory analyses revealed similarly high awareness rates for adults with ASD and TD for social stimuli at both low- and high-perceptual load levels, suggesting that in both groups, social stimuli were processed regardless of level of perceptual load.

The absence of group differences for awareness of a socially meaningful stimulus may however be the result of the specific load manipulations used. There is evidence to suggest that load effects on awareness are sensitive to age such that a higher level of perceptual load is required to modulate awareness in adults compared to children (Remington et al. 2014). Since Tyndall et al. (2018) used identical load manipulations compared to Tillmann et al. (2015) but sampled across a different age range (i.e. adults vs. children), the level of perceptual load may not have been high enough to modulate awareness of the social stimulus. Several other methodological limitations of these previous studies must also be noted. For example, participant’s performance on the central task was measured by line judgment accuracy (i.e. correct/incorrect line judgment), which provides only a coarse measure of performance. Since only those participants with accurate task performance on the critical trial were included in analyses, it remains unclear how performance differences between participants, particularly on the critical trial, might have affected awareness reports. For example, it may be conceivable that increased awareness rates in the ASD group are a consequence of them diverting attentional resources from the visual to the auditory modality. Measuring line judgment performance alone means such a possibility cannot be ruled out. Second, increased detection rates of the critical stimulus (CS, the unexpectedly presented stimulus) on the critical trial may be related to higher perception thresholds for perceiving the CS in some individuals, but individual perceptual thresholds of the CS were not measured. Third, the retrospective measure of awareness with a surprise question about an unexpected stimulus raises the possibility that a failure to report the presence of the auditory stimulus may reflect, in some cases, rapid forgetting. In the visual domain, such ‘inattentional amnesia’ has been described by Wolfe (1999) and refers to the idea that a failure to detect the presence of the unexpected stimulus might be related to a weak memory trace of the unexpected stimulus rather than inattention. Another possibility is that the findings reflect a change in the response criterion such that participants may be more reluctant to admit noticing an unexpected stimulus for which there is only a weak memory trace in conditions of high perceptual load. In summary, since a range of alternative explanations may account for these findings, the load-dependent effects on inattentional deafness for socially meaningful auditory stimuli vs. a neutral, non-social auditory stimuli in individuals with ASD remain poorly understood.

The current study attempted to take each of these concerns into account: First, the high load condition featured a higher load manipulation compared to previous studies (Tillmann et al. 2015; Tyndall et al. 2018). Second, each participant’s reaction time was measured across all trials to (a) obtain a more nuanced measure of task performance and (b) evaluate whether increased awareness rates in the ASD group are a consequence of them diverting attentional resources from the visual to the auditory modality. A shift in attention of participants away from the visual task to the auditory modality would likely be reflected in a significant difference between reaction time (RT) performance on the critical trial compared to average RTs for line judgement on non-critical trials. Third, participants performed an auditory threshold task after completing the inattentional deafness task. This will test whether the two groups (ASD vs. TD) differ in perceptual thresholds and will confirm whether the intensity level of the auditory stimulus is well above the threshold for each participant. Fourth, to lessen potential effects of rapid forgetting, the surprise question following the critical trial was presented after a short and fixed amount of time following the critical trial.

The improved task design of the current study allowed us to comprehensively assess the effect of perceptual load on attention to social and non-social auditory stimuli in individuals with ASD compared to matched TD controls. Our aim here is to further our understanding of the mechanisms of attention involved in processing of unexpected social auditory stimuli in ASD and to test more rigorously the effect of visual perceptual load on auditory awareness of neutral, non-social auditory stimuli. In line with previous findings of the reduced effects of perceptual load on non-social auditory stimuli processing in ASD (Tillmann et al. 2015; Tillmann and Swettenham 2017), it was hypothesised that increasing the perceptual load of a visual task would reduce awareness rates of a neutral non-social stimulus in TD, but not in individuals with ASD. In line with a growing body of research investigating the effects of load on attention to non-social distracting information in ASD (Remington et al. 2009; Remington et al. 2012a, b; Swettenham et al. 2014; Remington and Fairnie 2017; Tillmann and Swettenham 2019), this would be indicative of an increased perceptual capacity in ASD. Conversely, awareness rates for an unexpected speech sound were hypothesised to remain unaffected by load manipulations in TD individuals, i.e. awareness rates will remain high at high perceptual load. In individuals with ASD, we predicted that perceptual load has a similar effect on awareness rates regardless of whether the additional stimulus is social or non-social, suggesting that speech sounds are processed as if they are neutral sounds.

## Method

### Participants

63 adolescents with ASD and 62 typically developing (TD) adolescents took part in this study. Participants with ASD had a clinical diagnosis of ASD according to DSM-IV-TR (American Psychiatric Association 2000). In addition, parent-reported ASD symptomatology was measured using the *Social Communication Questionnaire* (SCQ; Rutter et al. 2003). Participants were excluded if they obtained a score of less than 9 correct on the 12 non-critical trials or made an incorrect line judgment on the critical trial. These exclusion criteria were necessary to make sure that all participants engaged with the primary task and followed task instructions (i.e. rather than responding randomly on the line discrimination task). It was particularly important to only include participants who had clearly engaged in the line-length discrimination (of either high or low load) while the irrelevant stimulus was presented. In addition, participants were excluded if they were unable to hear the target sound on the control trial. This final, full-attention trial was necessary to compare awareness rates with the critical, unattended trial to estimate the degree to which attention influences perception. Overall, these exclusion criteria resulted in 12 participants (8 ASD, 4 TD) being excluded from further analysis. Of those, five participants made an incorrect line judgment on the critical trial (4 ASD, 1 TD) and seven did not hear the critical stimulus (CS) on the control trial (4 ASD, 3 TD). The remaining 58 TD participants (33 males: 25 females) and 55 participants with ASD (49 males: 6 females) were matched for chronological age (*t*(111) = 1.85, *p* = 0.068) and non-verbal ability (*t*(111) = 1.10, *p* = 0.275) using the *Raven’s Standard Progressive Matrices* (Raven et al. 1998; see Table 1 for sample characteristics).

**Table 1:** Sample characteristics

Variable	Statistic	ASD (*N* = 55)	TD (*N* = 58)
Age (in years)	Mean (95% *CI*)	14.48 (14.17–14.78)	14.82 (14.61–15.03)
	*SD* (95% *CI*)	1.14 (0.96–1.40)	0.80 (0.68–0.98)
	Range	[11.10–17.45]	[11.64–15.83]
Non-verbal ability	Mean (95% *CI*)	43.69 (42.11–45.27)	44.88 (43.39–46.37)
	*SD* (95% *CI*)	5.84 (4.92–7.20)	5.66 (4.79–6.93)
	Range	[31–56]	[35–57]
SCQ Total	Mean (95% *CI*)	25.78 (24.27–27.29)	–
	*SD* (95% *CI*)	5.60 (4.71–6.89)	–
	Range	[17–35]	–

*95% CI* 95% confidence interval, *SD* standard deviation, *ASD* autism spectrum disorder, *TD* typically developing, *SCQ* social communication questionnaire; Non-verbal ability measured using the Raven’s Progressive Matrices

The design of this study is a 2 (‘group’: ASD vs. TD) × 2 (‘perceptual load condition’: high vs. low) × 2 (‘critical stimulus type’: social vs. non-social) design, with all participants being randomly allocated across perceptual load and critical stimulus (CS) conditions. Specifically, participants were distributed across four experimental conditions: (1) social CS at low load (13 ASD; 14 TD), (2) social CS at high load (14 ASD; 14 TD), (3) non-social CS at low load (14 ASD; 15 TD), and (4) non-social CS at high load (14 ASD; 15 TD). Each participant only completed one of the four experimental conditions, and thus completed a single critical trial, since it was important that participants were naïve to the aims of the experiment and were not expecting an additional stimulus or a surprise question. The sample size in each of the four experimental conditions (i.e. cells) is in line with previous studies using a similar experimental design (Swettenham et al. 2014; Tillmann et al. 2015; Tyndall et al. 2018). Informed consent was obtained from all individual participants included in the study.

### Stimuli

The experiment was created with Microsoft Visual Basic (version 6) and presented on an IBM Lenovo Thinkpad 14.1″ personal laptop (1440 × 900-pixel resolution). A viewing distance of 60 cm was maintained throughout the experiment. On each trial, a black cross (RGB: 0, 0, 0) centred at fixation was presented against a white background (RGB: 255, 255, 255), with either the horizontal (H) or the vertical (V) line of the cross being longer than the other one. The perceptual load of the line discrimination task was manipulated by increasing the visual angle of one of the arms of the target cross. In the high perceptual load condition, the short arm of the cross extended 3.35° and the long arm extended 3.9°. In the low perceptual load condition, the short arm extended 1.25° and the long arm extended 3.9°. Presentation of the cross was randomised across experimental trials and counterbalanced on the critical trial.

Auditory stimuli were prepared with Audition and SFSWin and played to participants through a pair of Sennheiser HD 25-1-II stereo headphones. Two target sounds were created: a male person saying ‘Hi’ (social stimulus) and a saw-tooth wave (non-social beep stimulus), and matched for pitch (85–150 Hz), duration (176 ms) and intensity level (33 dB). Sound pressure levels were measured using a Bruel and Kjaer 4153 artificial ear and an Ono Sokki CF-350Z spectrum analyser. The duration of the auditory stimuli was set at 176 ms to match the presentation time of the visual cross stimulus.

### Procedure

**Fig. 1:**
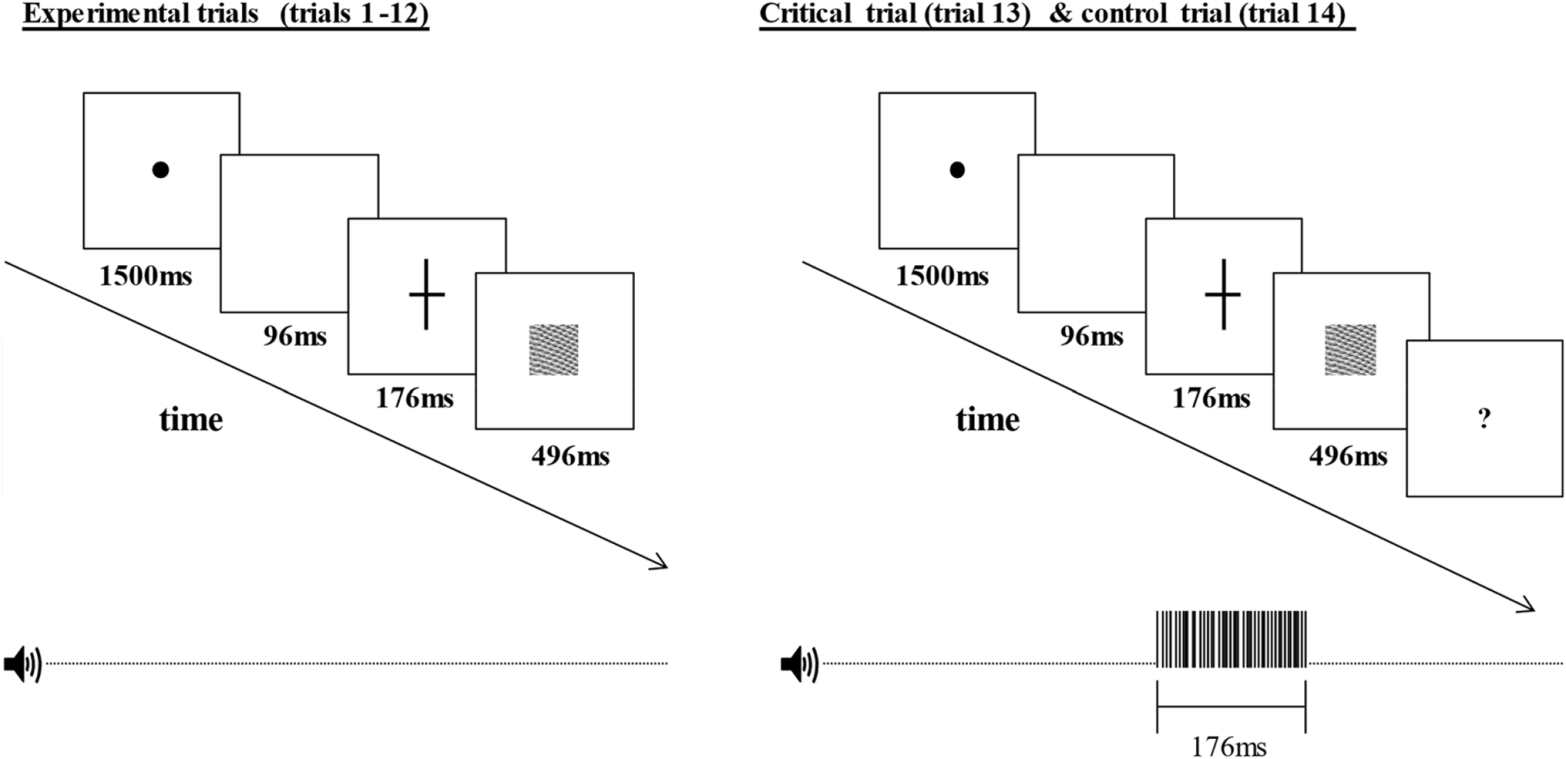
Experimental procedure

At the beginning of each trial, a black circle (0.15°) was presented at the centre of the screen (1500 ms), followed by a blank display (96 ms), a black cross (176 ms) and a visual mask (496 ms; see Fig. 1 for a graphical illustration). Participants made the cross task response (i.e. indicating which line of the cross was longer than the other one: horizontal or vertical) immediately after the black cross was displayed. A 2 s intertrial interval followed every response. Participants performed a total of 20 trials: 6 practice trials, 12 experimental trials, 1 critical trial and 1 control trial, and reaction time (RT) and accuracy data were recorded. There was no feedback on task performance except for practice trials.

On the critical trial, an auditory stimulus was played concurrently with the cross presentation. Responses to the cross task were recorded as in the previous trial, yet immediately after participants made the cross-task response, awareness of the critical stimulus (CS) was assed via an on-screen prompt, asking participants whether they noticed anything else. Since the prompt appeared immediately on the screen following the participant’s response, this limits concerns relating to rapid forgetting of the CS of the participant (Wolfe 1999). Participants gave verbal responses and were asked to provide additional details of the CS (i.e. imitate the ‘hi’ or beep stimulus). The critical trial was then repeated in a control trial, which measured awareness of the CS in absence of attention to the visual task. Prior to the control trial, participants were told to ignore the cross stimulus and instead attend to any other stimulus they might notice. Only those participants who successfully identified the CS on the control trial were included in further analyses.

After completing the line discrimination task, the perceptual threshold for the CS (i.e. beep or ‘Hi’ stimulus, depending on the task condition performed), was established for each participant. Using a two-alternative forced-choice (2AFC) adaptive threshold procedure, two consecutive correct detection responses resulted in a reduction in target sound intensity of 1 dB, making it more difficult to detect the target sound in the next trial. Conversely, one incorrect response increased target sound intensity by 1 dB, making it easier to detect the target sound in the next trial. Individual thresholds were estimated based on the average of five reversals (point at which direction is changed, i.e. either when producing a correct response followed by an incorrect response or when producing two correct responses after an incorrect response).

## Results

### Line Discrimination Performance

Statistical analyses were conducted using STATA software 15.0 (StataCorp 2017). Trials with reaction times (RTs) greater than 2500 ms were discarded. RTs were analysed using multiple regression, while task accuracy rates (value range: 9—12) were analysed using ordered logistic regression. Statistical models included as categorical predictor variables group (ASD vs. TD), stimulus type (neutral vs. social), task condition (low vs. high) and their interactions. For RTs, there was a significant effect of condition, with faster RTs in the low (*M* = 551.63, *SD* = 159.14, 95%CI [509.01; 594.25]) compared to the high perceptual load condition (*M* = 827.97, *SD* = 200.74, 95%CI [774.71; 881.24], *p* = 0.001, Cohen’s d = 1.52; see Supplementary Table 1 for full results). A similar pattern was also observed for error rates, with lower error rates in the low (*M* = 11.25, *SD* = 0.72, 95%CI [11.06; 11.44]) relative to the high perceptual load condition (*M* = 10.77, *SD* = 0.93, 95%CI [10.53; 11.02]), which was however not significant (*p* = 0.052, d = 0.58). There were no differences in RTs or task accuracy across main groups, stimulus types or tasks and no interaction terms were significant.

To evaluate whether differences in awareness rates between groups, conditions or tasks may be related a shift in attention away from the primary visual task on the critical trial, an RT difference score was created (RT_non-critical trials_ − RT_critical trial_), where a positive score reflects a slowing of response on the critical trial relative to average response times on non-critical trials. A multiple regression model was run on the RT difference score and included as categorical predictor variables group (ASD vs. TD), stimulus type (neutral vs. social), task condition (low vs. high) and their interactions. Results demonstrated no significant main effects of group (*p* = 0.968), stimulus type (*p* = 0.239) or condition (*p* = 0.707). Also, none of the two-way and three-way interaction terms were significant (all *p*’s < 0.154).

### Detection of the CS

**Fig. 2:**
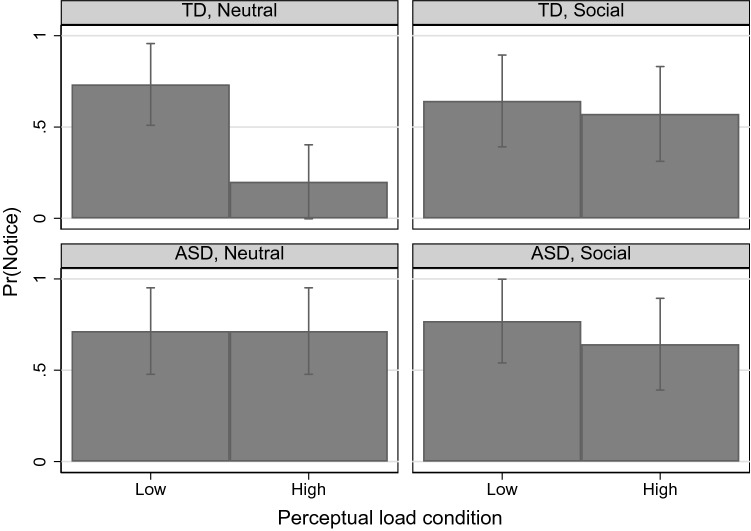
Average predicted probabilities of CS awareness (including 95% Confidence Interval) by task, load and diagnostic group

Logistic regression was used to predict detection rates of the CS and included as categorical predictor variables group (ASD vs. TD), stimulus type (neutral vs. social), task condition (low vs. high) and their interactions. Predicted probabilities are reported instead of odds ratios and were obtained with the margins command in STATA. Bonferroni-corrected *p*-values are reported for individual contrasts. Holding all other factors constant, there was a main effect of group, with individuals with ASD being 18% more likely than TD individuals to notice the critical stimulus (ASD = 71% vs. TD = 53%; x^2^(1) = 4.21, *p* = 0.04). Across diagnostic groups and task stimuli, all individuals were 19% more likely to report awareness in the low perceptual load compared to the high perceptual load condition (Low = 71% vs. High = 0.53%; x^2^(1) = 4.95, *p* = 0.026). No main effect of task was found (*p* = 0.43) and none of the two-way interactions were significant (i.e. Group × Condition: *p* = 0.148; Group × Task: *p* = 0.376; Task × Condition: *p* = 0.304). Importantly, the three-way interaction between Group, Task, and Condition was significant (x^2^(4) = 11.17, *p* = 0.025). Inspecting the three-way interaction (Fig. 2), it appeared that the effect of perceptual load on awareness for the two groups differed according to stimulus type (i.e. social or neutral stimulus).

To further investigate these relationships, post-hoc logistic regressions were run for each stimulus type separately and included main effects for group and condition, as well as their two-way interaction. When presented with a neutral auditory stimulus, there was a significant effect of group, with the ASD group showing higher awareness rates overall (ASD = 71% vs. TD = 47%; x^2^(1) = 4.64, *p* = 0.031). Across groups, participants reported greater awareness in low- compared to high-load conditions (Low = 71% vs. High = 0.53%; x^2^(1) = 5.79, *p* = 0.016). In addition, the two-way interaction between group and load condition was significant (x^2^(1) = 5.38, *p* = 0.02). Individual contrasts revealed that while perceptual load had no effect on awareness in children with ASD (x^2^(1) < 0.01, *p* > 0.9), TD children had significantly reduced awareness rates at high- compared to low perceptual load (High = 73% vs. Low = 20%; x^2^(1) = 12.00, *p* = 0.001). In conditions featuring the social auditory stimulus, no differences in awareness were found between groups (*p* = 0.437), load conditions (*p* = 0.437). The two-way interaction between group and load was also not significant (*p* = 0.711).

To account for the possibility that individual variation in perceptual thresholds influenced awareness rates and thus may explain the group differences observed, the analysis was re-run and included alongside all predictor variables above and their interactions also perceptual threshold data from each participant. Results confirmed previous significant findings of the main effects of group (*p* = 0.041) and task condition (*p* = 0.019). Importantly, the three-way interaction between Group, Task and Condition (x^2^(4) = 11.21, *p* = 0.024) also remained significant, with post-hoc analyses confirming the pattern of findings of reduced awareness at high perceptual load in TD individuals relative to individuals with ASD for the neutral, but not socially-relevant stimulus.

## Discussion

This study demonstrated that the effect of visual perceptual load on auditory awareness in individuals with ASD compared to TD controls differed according to the type of auditory stimulus presented (social or neutral stimulus). Awareness rates of an unexpected neutral, non-social auditory stimulus remained unaffected by increasing perceptual load in individuals with ASD yet dropped significantly in TDs. The pattern of findings, particularly in TD individuals, was notably different when a socially meaningful auditory stimulus was presented. Unlike awareness rates for the neutral stimulus that dropped as a function of perceptual load, awareness rates of the socially meaningful auditory stimulus remained high as perceptual load increased. This suggests that in TD individuals, processing of socially meaningful relative to non-social auditory stimuli proceeded regardless of the level of perceptual load. For individuals with ASD, we found that increasing perceptual load did not affect awareness rates of the social stimulus.

The reduced effect of visual perceptual load on auditory awareness for a neutral sound in individuals with ASD is consistent with the hypothesis that individuals with ASD have an increased perceptual capacity that operates across sensory modalities (Tillmann et al. 2015; Tillmann and Swettenham 2017, 2019; Tyndall et al. 2018). This interpretation follows from the application of Load theory. Load theory proposes that conscious perception of task-irrelevant stimuli depends on whether the level of perceptual processing (i.e. perceptual load) of a task exhausts perceptual capacity of an individual. If a task has a high enough perceptual load, all available capacity is consumed, and task-irrelevant stimuli are not perceived—inattentional deafness occurs. Conversely, a task with low load is unlikely to exhaust an individual’s full capacity, resulting in an automatic and involuntary ‘spill-over’ of attentional resources to the processing of task irrelevant stimuli—the additional stimulus is perceived. Thus, the level of perceptual load at which task-irrelevant stimuli are no longer processed relates to an individual’s perceptual capacity. As awareness rates for the neutral sound remained unaffected by the increase in perceptual load in individuals with ASD, yet were significantly reduced in TD individuals (matched in age and non-verbal ability scores), the results suggest that individuals with ASD had processing resources left-over to also attend to the neutral sound, whereas TD individuals did not. This provides further support for an increased perceptual capacity in ASD and in line with findings of a reduced effect of perceptual load on selective attention of neutral, non-socially meaningful stimuli and resulting in enhanced processing of extraneous information relative to TD participants under high load conditions (Remington and Fairnie 2017; Swettenham et al. 2014; Remington, Swettenham, et al. 2012a, b; Remington et al. 2009).

Importantly, the current study allowed for a more comprehensive assessment of cross-modal perceptual load effects on auditory awareness in ASD by employing an improved experimental design compared to previous studies (Tillmann et al. 2015; Tyndall et al. 2018). In addition to measuring error rates per trial, we obtained participant’s reaction time to evaluate whether a trade-off in task performance on the critical trial may account for the differences in awareness rates across diagnostic groups. Across task conditions, diagnostic groups and their interaction, we found no evidence of a slowing of response on the critical trial relative to average response times on non-critical trials, suggesting that greater awareness for the neutral stimulus on the critical trial in the ASD group is unlikely to be related to individuals with ASD investing fewer attentional resources on the visual task during the critical trial. By measuring perceptual thresholds of the CS across participants, we demonstrated that group differences in perceptual thresholds are unlikely to account for the pattern of results observed. This is important, since sensory atypicalities, particularly in the auditory modality, have often been reported in individuals with ASD (Robertson and Baron-Cohen 2017). Lastly, to lessen potential effects of rapid forgetting, the surprise question following the critical trial was presented after a short and fixed amount of time following the critical trial. Although we cannot completely discount the possibility that a weak memory trace of the unexpected stimulus rather than inattention accounts for the pattern of findings observed in conditions of high perceptual load, the fact that the surprise question was presented after a fixed time for both groups goes some way to control for this limitation.

While our findings suggest superior perceptual processing in ASD, in line with theories of enhanced local processing (Happé and Frith 2006; Mottron et al. 2006), there are studies to suggest that perceptual processing is impaired in ASD (Haigh et al. 2015), and particularly multisensory integration (Stevenson et al. 2014; Noel et al. 2018; Crosse et al. 2019). However, it is important to point out that work on multisensory integration in ASD differs considerably in terms of task requirements from an inattentional deafness task used here: the former typically results in enhanced perception of one stimulus in the presence of a stimulus in a different modality, while the latter is a selective attention task in which the additional auditory CS does not facilitate processing of the visual stimulus, or vice versa. The aim of an inattentional deafness task is thus to examine the degree to which attention influences conscious perception.

The current study demonstrated that for TD individuals an unexpected, yet ecologically salient speech sound captures attention regardless of the level of visual perceptual load, whereas a neutral sound does not. Considering the special biological and social significance of speech sounds could explain these findings. It may be adaptive that socially meaningful auditory information, unlike other neutral information, is processed irrespective of the level of visual perceptual load. Even if unexpected auditory information is not relevant for current task behaviours, as was the case in the current study, it can potentially carry important information including social cues (e.g. information on an individual’s affect), which may be detrimental not to attend to. Note that the non-social critical stimulus was matched as closely as possible to the social stimulus on a number of acoustic properties including pitch, duration and intensity, something which was not done by previous studies (Tyndall et al. 2018). Thus, whilst both stimuli had similar complex acoustic properties, the social stimulus retained its ‘speechness’ quality relative to the non-social stimulus. Any differences in awareness are therefore unlikely to be related to differences between the two sound stimuli in basic acoustic qualities.

Individuals with ASD showed a pattern of both typical performance under load for the social CS and enhanced performance under load compared to TD controls for the non-social CS. The critical difference to the TD results is however that since awareness rates for the social and non-social stimulus were similarly high at high perceptual load, we cannot conclude that the social stimulus was ‘special’ for individuals with ASD. It could be that in individuals with ASD, social auditory information captures attention similarly as socially neutral information, i.e. individuals with ASD treat social and non-social information in a similar manner. This may have important developmental implications, since an absence of preference for social sounds over non-social sounds may restrict the child’s exposure to typical social interactions and affecting subsequent development of higher-order social communicative functions. Alternatively, individuals with ASD also process socially meaningful information regardless of level of load—similar to TD controls. In the absence of any differences under high perceptual load in awareness of the neutral CS compared to the social CS we are however unable to un-pick these questions further. It is a notable limitation of the current study that participants did not perform a very high load task condition. To further explore these issues, it would be interesting to measure the effect of visual perceptual load on awareness of a social vs. non-social stimulus in ASD at very high levels of perceptual load to see if there is a point at which a non-social stimulus CS does not reach awareness while a social stimulus does, or whether both types of stimuli suffer the same fate of not being noticed at higher levels of load. In addition, the one-trial nature of the inattentional deafness paradigm did not allow us to calculate more nuanced measures of CS detection performance including detection sensitivity and response bias. Future research could use previously established signal detection paradigms in ASD (Tillmann and Swettenham 2017, 2019) to contrast awareness of a non-social auditory CS compared to a social auditory CS in a repeated within-participant design.

## Electronic supplementary material

Below is the link to the electronic supplementary material.

Supplementary file1 (DOCX 17 kb)
