# Supplementary material for: The Effect of Visual Perceptual Load on Auditory Awareness of Social vs. Non-social Stimuli in Individuals with Autism
Source: J Autism Dev Disord. 2020 Jul 1;51(4):1028–38. doi: 10.1007/s10803-020-04587-0 (PMC7985111; doi:10.1007/s10803-020-04587-0)
Supplement: Supplementary file 1 — Supplementary file1 (DOCX 17 kb) [file 10803_2020_4587_MOESM1_ESM.docx]

**Supplementary Table 1** Main and interaction effects of group, condition and task on reaction time, error rate and auditory perceptual thresholds

|  | Reaction Time (in msec) | | |  | Error rate | | |  | Perceptual threshold (in db) | | |
| --- | --- | --- | --- | --- | --- | --- | --- | --- | --- | --- | --- |
| Variable | *b*  *SE (b)* | *t* | 95% *CI* |  | *b*  *SE (b)* | *t* | 95% *CI* |  | *b*  *SE (b)* | *t* | 95% *CI* |
| Group (ASD vs. TD) | 52.67 | 0.79 | [-78.98,184.32] |  | 0.26 | 0.39 | [-1.06,1.59] |  | 1.45 | 0.82 | [-2.07,4.97] |
|  | (66.39) |  |  |  | (0.68) |  |  |  | (1.78) |  |  |
| Condition (Low vs. High) | 215.83 | 3.31^**^ | [86.47,345.19] |  | -1.32 | -1.94^(*)^ | [-2.65,0.01] |  | 0.93 | 0.53 | [-2.53,4.39] |
|  | (65.24) |  |  |  | (0.68) |  |  |  | (1.74) |  |  |
| Task (Neutral vs. Social) | 15.59 | 0.17 | [-170.59,201.77] |  | -0.48 | -0.70 | [-1.82,0.86] |  | 0.17 | 0.09 | [-3.35,3.69] |
|  | (93.90) |  |  |  | (0.68) |  |  |  | (1.78) |  |  |
| Group X Condition | -19.83 | -0.30 | [-151.47,111.82] |  | 0.78 | 0.79 | [-1.14,2.70] |  | -1.15 | -0.46 | [-6.13,3.83] |
|  | (66.39) |  |  |  | (0.98) |  |  |  | (2.51) |  |  |
| Group X Task | 6.65 | 0.07 | [-182.95,196.26] |  | 0.69 | 0.69 | [-1.28,2.66] |  | -3.34 | -1.31 | [-8.41,1.73] |
|  | (95.62) |  |  |  | (1.01) |  |  |  | (2.56) |  |  |
| Condition X Task | 85.39 | 0.91 | [-100.79,271.57] |  | 0.39 | 0.41 | [-1.50,2.29] |  | -4.50 | -1.79 | [-9.48,0.47] |
|  | (93.90) |  |  |  | (0.97) |  |  |  | (2.51) |  |  |
| Group X Condition X Task | 42.83 | 0.32 | [-224.03,309.68] |  | -1.25 | -0.87 | [-4.04,1.55] |  | 2.46 | 0.68 | [-4.68,9.60] |
|  | (134.58) |  |  |  | (1.43) |  |  |  | (3.60) |  |  |

Note: *b* = regression coefficient, *SE(b)* = Standard Error of regression coefficient, t = t-statistic, 95% *CI* = 95% Confidence Interval of regression coefficient; ASD = Autism Spectrum Disorder; TD = Typically Developing; (*) *p* <.1; * *p* <.05; ** *p* <.01
